# Expression of midkine in the early stage of carcinogenesis in human colorectal cancer

**DOI:** 10.1038/sj.bjc.6690030

**Published:** 1999-09-24

**Authors:** C Ye, M Qi, Q-W Fan, K Ito, S Akiyama, Y Kasai, M Matsuyama, T Muramatsu, K Kadomatsu

**Affiliations:** Department of Pathology, Fujita Health University School of Medicine, Toyoake, Aichi 470-1192, Japan; Department of Biochemistry, Nagoya University School of Medicine, Showa-ku, Nagoya, 466-8550; Department of Surgery II, Nagoya University School of Medicine, Showa-ku, Nagoya, 466-8550, Japan

**Keywords:** carcinogenesis, colorectal adenoma, colorectal carcinoma, midkine, pleiotrophin

## Abstract

It has been suggested that a heparin-binding growth factor, midkine (MK), plays an important role incarcinogenesis because of its frequent overexpression in various malignant tumours. To clarify whether or not MK contributes to theearly stage of carcinogenesis, we examined the status of MK mRNA in 20 adenomas with moderate- and severe-grade dysplasia, 28carcinomas and 28 corresponding normal tissues, by means of Northern blotting. The MK expression level was significantly moreelevated in adenomas than in normal tissues *P*< 0.001, unpaired Student's *t* -test). A difference wasalso observed between carcinomas and the corresponding normal tissues *P*< 0.04, paired Student's *t*-test). Moreover, MK immunostaining was positive in the adenomas with moderate- and severe-grade dysplasia and in the carcinomas,but not in mild-grade dysplasia or in normal tissues. These findings were in line with those on Western blotting. In three patientswith both adenomas with moderate- or severe-grade dysplasia and carcinomas, elevated MK expression was observed in the neoplasticlesions. This is the first report of the association of elevated MK expression with the early stage of carcinogenesis in humans. © 1999 Cancer Research Campaign


*British Journal of Cancer* (1999) **79**(1), 179–184
© 1999 Cancer Research Campaign

### Expression of midkine in the early stage of carcinogenesis in human colorectal cancer

**C Ye[1], M Qi[2], Q-W Fan[2], K Ito[3], S Akiyama[3], Y Kasai[3], M Matsuyama[1], T Muramatsu[2] and K Kadomatsu[2]**

[1]Department of Pathology, Fujita Health University School of Medicine, Toyoake, Aichi 470-1192, Japan; [2]Department of Biochemistry and [3]Department of Surgery II, Nagoya University School of Medicine, 65 Tsurumai-cho, Showa-ku, Nagoya 466-8550, Japan

**Summary** It has been suggested that a heparin-binding growth factor, midkine (MK), plays an important role in carcinogenesis because of its frequent overexpression in various malignant tumours. To clarify whether or not MK contributes to the early stage of carcinogenesis, we examined the status of MK mRNA in 20 adenomas with moderate- and severe-grade dysplasia, 28 carcinomas and 28 corresponding normal tissues, by means of Northern blotting. The MK expression level was significantly more elevated in adenomas than in normal tissues ($P < 0.001$, unpaired Student's *t*-test). A difference was also observed between carcinomas and the corresponding normal tissues ($P < 0.04$, paired Student's *t*-test). Moreover, MK immunostaining was positive in the adenomas with moderate- and severe-grade dysplasia and in the carcinomas, but not in mild-grade dysplasia or in normal tissues. These findings were in line with those on Western blotting. In three patients with both adenomas with moderate- or severe-grade dysplasia and carcinomas, elevated MK expression was observed in the neoplastic lesions. This is the first report of the association of elevated MK expression with the early stage of carcinogenesis in humans.

**Keywords:** carcinogenesis; colorectal adenoma; colorectal carcinoma; midkine; pleiotrophin

In the last few years, many studies have confirmed that growth factors not only promote tissue proliferation but also induce malignant transformation (Cross and Dexter, 1991). Over-expression of growth factors has been found in many human tumours, and this phenomenon is often considered to be a cause of carcinogenesis (Aaronson, 1991). MK is a product of a retinoic acid-responsive gene and belongs to the new family of heparin-binding growth/differentiation factors (Kadomatsu et al, 1988, 1990; Tomomura et al, 1990*a*, *b*; Muramatsu, 1993, 1994). It plays a role in neuronal survival and differentiation (Michikawa et al, 1993; Muramatsu et al, 1993; Unoki et al, 1994) and is also expressed at higher levels in human tumours, such as Wilms' tumours (Tsutsui et al, 1993), lung carcinomas (Garver et al, 1993), breast carcinomas (Garver et al, 1994), neuroblastomas (Nakagawara et al, 1995), hepatocellular, gastric, pancreatic and colon carcinomas (Aridome et al, 1995) and urinary bladder carcinomas (O'Brien et al, 1996), than in corresponding normal tissues. However, it remains unknown whether MK plays a role in either an early stage of carcinogenesis or a later stage. The progression from colorectal neoplasia to an invasive cancer provides an excellent opportunity for studying the status of MK, because most colorectal carcinomas appear to arise from adenomas. In the present study, we attempted to clarify the role of MK in carcinogenesis through examination of its expression in adenomas with different grades of dysplasia and in carcinomas at various stages by means of Northern blotting, Western blotting and immunostaining.

*Received 9 September 1997*
*Revised 1 April 1998*
*Accepted 14 May 1998*

*Correspondence to:* K Kadomatsu

### MATERIALS AND METHODS

### Sample collection

Fifty-six adenoma specimens were obtained from 46 patients (16 with mild-, 34 with moderate- and six with severe-grade dysplasia) who had undergone polypectomy at the Nogaki Hospital of Disease of the Anus and Rectum in Nagoya, Japan, from January 1997 to February 1998. Two or more specimens per case were available from nine patients, i.e. two adenomas with mild- and moderate-grade dysplasia in six patients, two adenomas with moderate-grade dysplasia in two patients and three adenomas with two moderate- and one severe-grade dysplasia in one patient. Paired specimens of carcinomas and corresponding normal tissues over 4 cm from the carcinoma lesions were collected from 32 patients (six stage A, 14 stage B and 12 stage C) who had not received any form of chemotherapy and radiation and who had undergone colorectal surgery at the Department of Surgery II, Nagoya University School of Medicine, between September 1996 and June 1997. There was no overlap between the above-mentioned adenoma patients and carcinoma patients. In addition, colorectal carcinoma, adenoma and normal tissue specimens in the same individual were obtained from three patients. The adenomas were graded according to the degree of dysplasia by means of histopathology, and the colorectal carcinomas were staged according to the Astler–Coller modification of Dukes' classification (Aster and Coller, 1954).

The tissues were obtained during surgery. Each specimen, if of sufficient volume, was divided into two parts, except for routine pathological examination with haematoxylin and eosin. One part was quickly frozen in liquid nitrogen and stored at –80°C until RNA extraction using guanidium thiocyanate, followed by ultra-centrifugation in a caesium chloride solution and/or until protein extraction and heparin–Sepharose column chromatography. The other part was fixed using 4% paraformaldehyde in 0.1 M

Dulbecco's phosphate-buffered saline (PBS) (pH 7.2) for about 16 h at 4°C. Paraffin blocks were then made for sectioning for MK immunostaining.

Because of the limited number of specimens, the numbers used for analyses were as follows: adenomas, 20 for Northern blot analysis, 50 for immunostaining and five for Western blot analysis (in other words, three for Northern, Western and immunostaining, two for Northern and Western, 11 for Northern and immunostaining, four for Northern and 36 for immunostaining); paired carcinoma and corresponding normal tissue specimens, 28 pairs for Northern blot analysis, 22 pairs for immunostaining and 14 for Western blot analysis (eight pairs for Northern, Western and immunostaining, four pairs for Northern and Western, 11 pairs for Northern and immunostaining, one pair for Western and immunostaining, five pairs for Northern, two pairs for immunostaining and one pair for Western); and carcinoma, adenoma and normal tissue samples from the same individuals, two for Northern blot analysis and immunostaining, and one for Northern and Western blot analyses as well as immunostaining.

### Northern and Western blot analysis

Northern blot analysis was performed as described previously (Kadomatsu et al, 1993). A 487-bp human MK cDNA fragment (nucleotide numbers 76–562) (Tsutsui et al, 1993) or a 3-kbp human β-actin cDNA (Nakajima-Iijima et al, 1993) was used as a probe. After appropriate exposure of the autoradiograms, the signal intensity was determined with computer software, NIH image.

For protein extraction, tissues, 100 mg each, were homogenized in ten volumes of ice-cold lysis buffer (10 mM Tris HCl, pH 7.6, 1% Triton X-100, 0.2 M sodium chloride), and then centrifuged at 10 000 ***g*** for 20 min at 4°C. One hundred microlitres of 10% heparin–Sepharose was added to the supernatant, followed by rotation overnight at 4°C. After washing three times with the lysis buffer and three times with the washing buffer (10 mM Tris HCl, pH 7.6, 0.2% Triton X-100, 0.5 M sodium chloride), protein samples (each corresponding to 40 mg of tissue) were subjected to SDS-polyacrylamide gel electophoresis as described by Laemmli (1970), and then transferred to a nitrocellullose membrane. The MK protein was detected with rabbit anti-human MK antibodies (dilution, 1:3000) (provided by Drs S. Ikematsu and S. Sakuma, Meiji Cell Technology Center) and horseradish peroxidase-labelled anti-rabbit IgG (Jackson Laboratory; dilution, 1:5000), with the use of an ECL system (Amersham).

### Immunohistochemistry

Paraffin sections (4 μm) were sequentially incubated at room temperature as follows: (1) with 10% skim milk to remove possible background for 20 min; (2) with affinity-purified rabbit anti-mouse MK antibodies at its working dilution (1:200) for 1 h; (3) with 0.3% hydrogen peroxide in methanol for 30 min to block endogenous peroxidase activity; (4) with the secondary antibodies (biotinylated anti-rabbit IgG; Southern Biotechnology Associates, Birmingham, AL, USA) for 30 min; (5) with avidin–horseradish peroxidase (Vector, Burlingame, CA, USA) for 30 min; and (6) with 3,3-diaminobenzidine containing hydrogen peroxide for 5 min for development. Washing was performed three times with PBS buffer (pH 7.4) after each incubation. The nuclei were stained with 1% methyl green (pH 4.0). The primary antibodies were replaced with PBS buffer as a negative control.

### RESULTS

Twenty adenomas (16 with mild- and four with severe-grade dysplasia) and 28 pairs of carcinomas and corresponding normal tissues were examined for MK RNA expression by means of Northern blotting. Representatives are shown in Figure 1A. On comparison of the densities of the autoradiograms with β-actin, the mean ratio of MK mRNA was found to be 1.35 (SD = 0.9) for normal colorectal tissues, but 3.72 (SD = 2.4) for adenomas. Since normal tissue corresponding to an adenoma was very difficult to obtain on polypectomy, we used the value for normal colorectal tissues from independent individuals with carcinomas as a relative standard. There was no statistical difference in age and gender

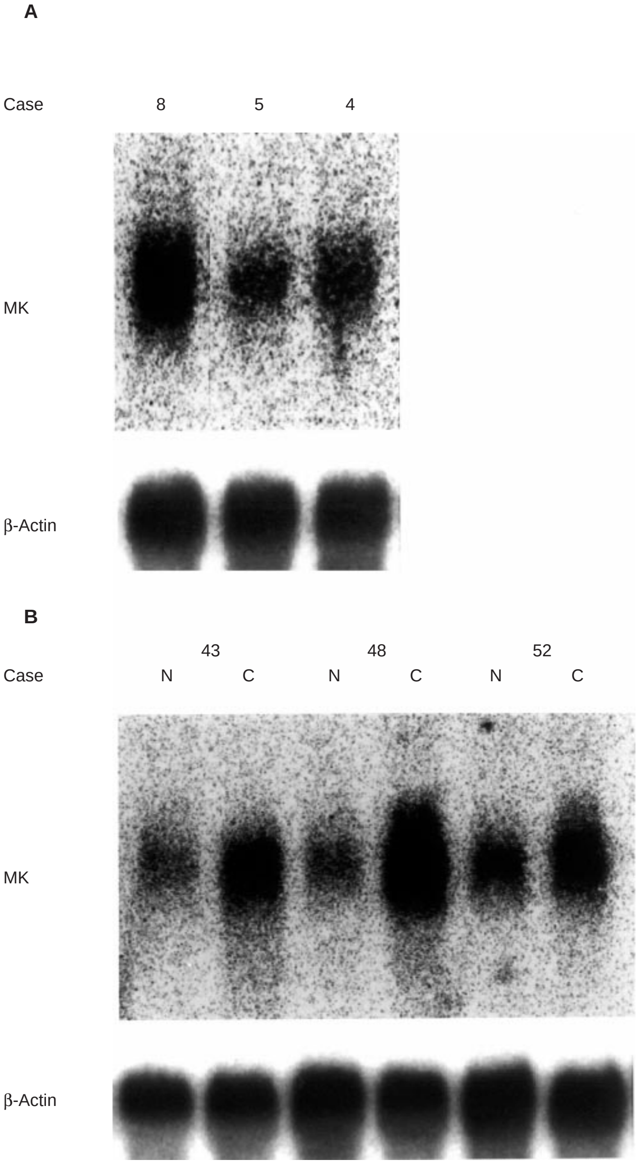

**Figure 1** MK RNA expression in colorectal adenomas and carcinomas. (**A**) MK mRNA overexpressed in colorectal adenomas. Cases 4 and 5 had tubular and villous adenomas with moderate-grade dysplasia, respectively, and case 8 had a tubular adenoma with moderate-grade dysplasia. (**B**) increased MK expression in colorectal carcinomas compared with in the corresponding normal tissues. Case 43 had a colorectal carcinoma at stage A, and cases 48 and 52 had colorectal carcinomas at stage C. N, normal tissue; C, carcinoma

*© Cancer Research Campaign 1999*

between the adenoma and carcinoma patients. A statistical difference was found between adenomas and normal tissues ($P < 0.001$) using the unpaired Student's *t*-test (Figure 2).

When investigating the status of MK mRNA in normal tissue and carcinoma samples, we often observed higher expression of MK in a carcinoma tissue than in the corresponding normal tissue, as shown in Figure 1B. A higher level of MK expression was observed in 19 of the 28 carcinoma samples (68%) than in the corresponding normal tissues, a statistically significant difference ($P = 0.036$) being revealed with the paired Student's *t*-test (Figure 2). Thus, the level of MK mRNA was elevated from the adenoma with dysplasia stage to the advanced carcinoma stage. In addition, interestingly, the MK mRNA expression level in adenomas was rather higher than that in carcinomas ($P < 0.01$, unpaired Student's *t*-test.

To determine the status of MK at the protein level during the progress of colorectal carcinogenesis, immunostaining was performed for 50 adenoma specimens, comprising 16 with mild-grade dysplasia, 29 with moderate-grade dysplasia and five with severe-grade dysplasia, and for 22 paired samples of carcinomas at different stages and corresponding normal tissues. MK staining was observed in adenomas with moderate- and severe-grade dysplasia and in carcinomas, but not in the normal colorectal mucosa or adenomas with mild-grade dysplasia (Figure 3 and Table 1). The intracellular distribution of MK appeared to be mainly in the cytoplasm, but also in some nuclei. Positive staining was observed in none of the 16 adenomas with mild-grade dysplasia, in 18 of the 29 with moderate-grade dysplasia, in all five with severe-grade dysplasia (Table 1) and in all 22 carcinomas (data not shown). The extent of positive staining paralleled the severity of the dysplasia.

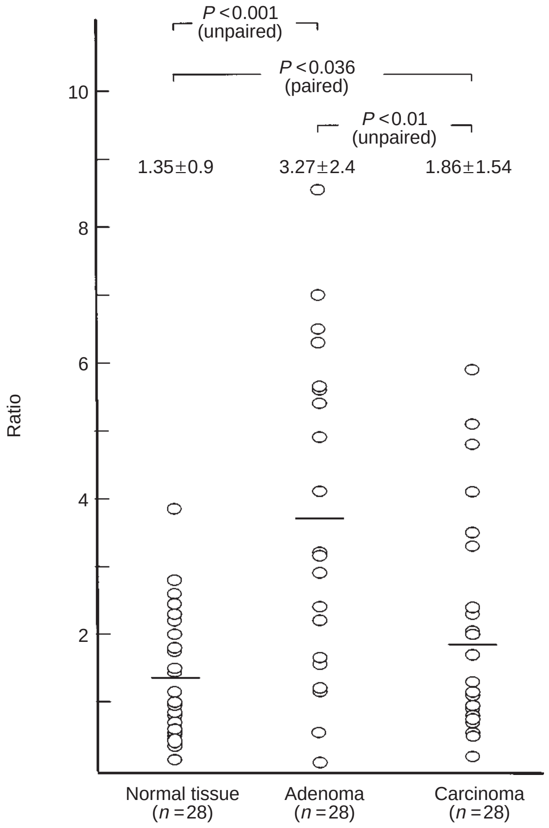

**Figure 2** Levels of MK mRNA in colorectal normal tissues, adenomas and carcinomas. The MK mRNA level was determined by means of NIH Image software with the β-actin gene as an internal control. The ratio of MK to β-actin in each sample was calculated. Bars represent averages. For statistical analysis, the unpaired and paired Student's t-tests were used

To confirm the results of immunostaining, Western blot analysis was performed. The protein could be extracted from two adenomas with severe-grade dysplasia, three with moderate-grade dysplasia and 14 paired specimens of carcinomas and corresponding normal tissues. In addition, samples from one case, in which adenoma with moderate-grade dysplasia, carcinoma at stage B and corresponding normal tissues were available, were also subjected to Western blot analysis. The level of the MK protein was much higher in carcinomas than in the corresponding normal tissues in all 14 paired specimens (examples are shown in Figure 4). Strong expression of the MK protein was also detected in all five adenomas (an example is shown in Figure 4). MK migrates as an approximately 17-kDa molecule; thus, the faint signals higher than this may be artifacts. Importantly, although only one case with adenoma, carcinoma and corresponding normal tissue was available for Western blot analysis, MK protein expression was much higher in the adenoma and carcinoma than in the corresponding normal tissue (Figure 4).

In three cases, normal, adenoma with moderate/severe-grade dysplasia and carcinoma tissues were all available for Northern blot analysis and immunohistochemistry. These cases showed that, in the same individual, MK RNA expression was elevated from the adenoma stage to the carcinoma stage, and MK protein expression revealed by immunohistochemistry exhibited a similar profile (Table 2). These findings were consistent with the MK expression profile observed in the mass studies described above.

### DISCUSSION

Carcinogenesis is a multistep process requiring, first, that the normally interdependent systems controlling proliferation and differentiation are uncoupled, and, second, that proliferation is stimulated in such a way as to result in extensive growth of the transformed cells. We recently demonstrated that MK has oncogenic potential because it induces the transformation of NIH3T3 cells and tumours in nude mice (Kadomatsu et al, 1997). Consistent with this finding, MK expression was observed from the precancerous lesion stage to the terminal carcinoma stage during carcinogenesis in hepatic carcinomas induced by diethyl-

**Table 1** Relationship between MK expression and the histopathology of colorectal adenomas

| Histopathology | Mild-grade dysplasia | Moderate-grade dysplasia | Severe-grade dysplasia |
|---|---|---|---|
| Morphology | | | |
| Tubular | 0/16 | 12/21 | 1/1 |
| Tubulovillous | 0 | 6/8 | 3/3 |
| Villous | 0 | 0 | 1/1 |
| Size | | | |
| <1 cm | 0/16 | 12/20 | 0 |
| 1–2 cm | 0 | 6/9 | 4/4 |
| >2 cm | 0 | 0 | 1/1 |

Denominator, number of samples analysed using immunostaining, numerator, number positive

© Cancer Research Campaign 1999

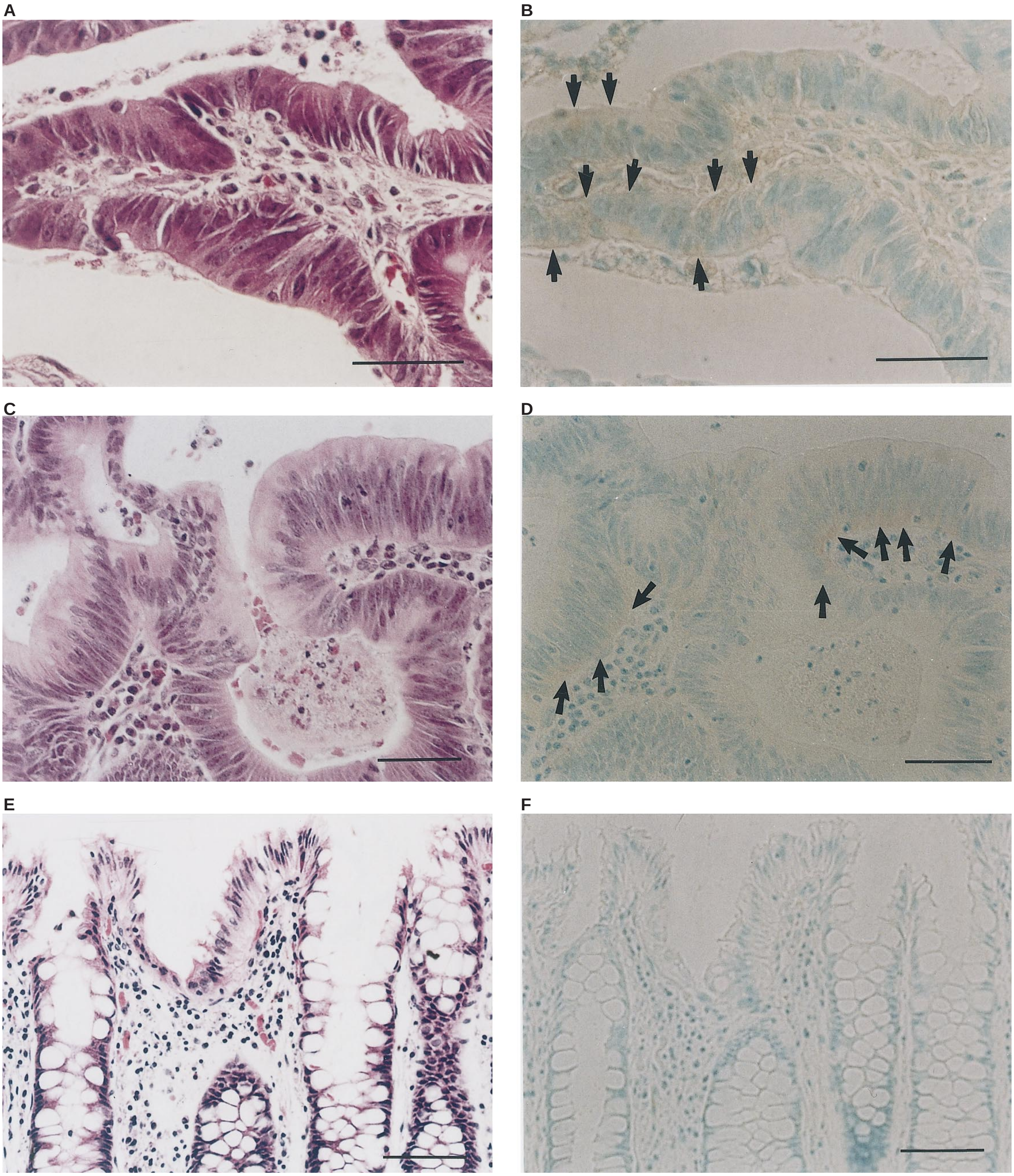

**Figure 3** MK immunostaining in colorectal neoplastic tissues. MK staining can be observed in carcinoma nests (arrows in **B**), and some lesions of adenoma with severe-grade dysplasia (arrows in **D**), but not in normal colorectal tissue (**F**). (**A**), (**C**) and (**E**) show haematoxylin and eosin staining corresponding to (**B**), (**D**) and (**F**) respectively. Bars 50 μm nitrosamine in rats (Kanda et al, manuscript in preparation). In the present study, we showed the following: (1) strong expression of MK mRNA appeared at the stage of adenomas with moderate/severe-grade dysplasia in human colorectal carcinogenesis; (2) the MK protein was also detected in the neoplastic tissues at the same stages; and (3) the extent of MK-positive staining increased according to the severity of dysplasia in human colorectal adenomas. In some cases, the changes seen in the levels of RNA expression were not very profound. Thus, we cannot exclude the possibility that these changes reflect a lower state of differentiation of the cells or an increased growth fraction, and thus have little to do with malignant transformation. But we

*© Cancer Research Campaign 1999*

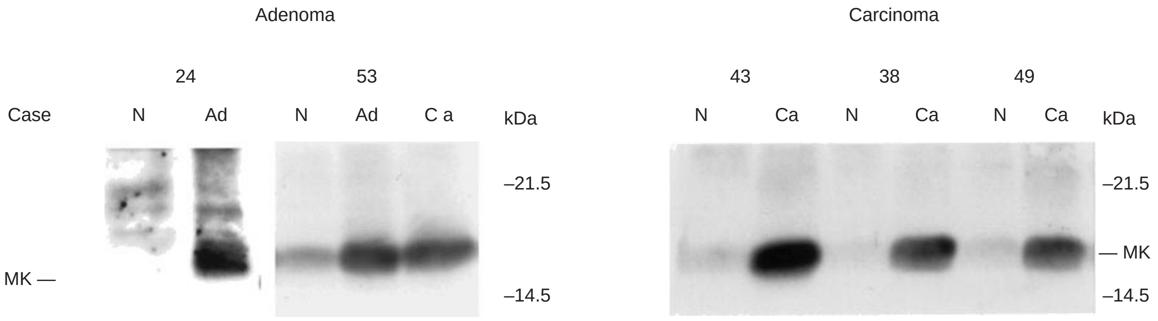

**Figure 4** MK in colorectal tissues, as analysed by Western blotting. Case 24, tubular villous adenoma with severe-grade dysplasia; case 43, colorectal carcinoma at stage A; case 38, stage B; and case 49, stage C. For case 53, both adenoma, with moderate-grade dysplasia, carcinoma, at stage B and corresponding normal tissues were available. The level of MK protein expression was much higher in adenomas and carcinomas than in normal tissues.

**Table 2** MK expression in colorectal tissues from the same individuals

| Case | Histopathology | Normal tissue | Adenoma | Carcinoma |
|---|---|---|---|---|
| 47 | Carcinoma in stage A and adenoma with severe-grade dysplasia | 2[a] (–)[b] | 3.2 (+) | 3.3 (+) |
| 53 | Carcinoma in stage C and adenoma with moderate-grade dysplasia | 2.8 (–) | 4.1 (+) | 5.1 (+) |
| 54 | Carcinoma in stage C and adenoma with moderate-grade dysplasia | 2.3 (–) | 2.9 (+) | 5.9 (+) |

[a]The ratio of MK to β-actin in each sample was calculated as described in Figure 2. [b]The results of immunostaining are shown in parentheses: (–), negative; (+), positive.

usually observed clearly large differences, as shown in Figure 4, between normal tissues and neoplastic tissues, including adenomas with moderate-severe-grade dysplasia and carcinomas, in protein expression on Western blot analysis. These findings suggest the importance of MK in the early stage of carcinogenesis in humans. This was further supported by the finding that MK expression was elevated at the RNA level, as well as the protein level, from the adenoma stage in the individuals for whom normal, adenomatous and carcinomatous colorectal tissues were subjected to examination of MK expression (Table 2 and Figure 4).

On the other hand, MK is frequently expressed at a high level in advanced human carcinomas. In the present study, we confirmed this in human colorectal carcinomas, consistent with the previous data reported by Aridome et al (1995). Concerning the possible involvement of MK in late stages of carcinogenesis, Choudhuri et al (1997) reported the angiogenic activity of MK. In addition, a positive relationship between the MK level and the clinical stages of neuroblastomas and urinary bladder carcinomas has been reported (Nakagawara et al, 1995; O'Brien et al, 1996).

To account for the importance of the growth factor in tumour development, the autocrine hypothesis has classically been proposed (Sporn and Todaro, 1980). Although the original concept was too restrictive and autocrine growth factor secretion also functions in normal growth regulation (Cross and Dextor, 1991; Daughaday and Deuel, 1991), autocrine or paracrine circuits in normal growth can also stimulate the growth of neighbouring oncogene-activated cells, and then the oncogene-activated cells may obtain a growth advantage by maintaining local critical concentrations of some growth factors (Dawson and Wynford-Thomas, 1995). There have been many reports on association of the expression of growth factors and early carcinogenesis. Insulin-like growth factor II is expressed in precancerous altered hepatic foci in woodchuck hepatitis virus carriers (Yang et al, 1993). Transforming growth factor α is expressed in precancerous altered hepatic foci in a rat hepatocarcinogenesis model involving initiation with diethylnitrosamine and promotion with phenobarbital, and the frequency of its expression is increased by the administration of a progressor agent such as ethylnitrosourea (reviewed by Pitot et al, 1996). Basic fibroblast growth factor expression is associated with squamous carcinogenesis of the head and neck in man, the expression being detected as early as in carcinomas in situ (Hughes et al, 1994). Furthermore, constitutive expression of transforming growth factor α in transgenic mice accelerates carcinogenic responses to chemical inducers (Tamano et al, 1994) and also increases the incidence of pancreas and liver cancers in cooperation with constitutively expressed c-*myc* (Sandgren et al, 1993). In an in vitro cell culture, MK is undoubtedly an autocrine growth factor for G401 cells, a Wilms' tumour cell line expressing abundant MK, because neutralizing antibodies for MK inhibit G401 cell growth (Muramatsu et al, 1993). MK also promotes the proliferation of several cell lines (Muramatsu and Muramatsu, 1991; Nurcombe et al, 1992). Accumulating evidence, including the present report, supports the autocrine hypothesis for tumour development, especially in terms of the mechanism by which oncogenic-activated cells achieve autonomous growth in early carcinogenesis.

With regard to the regulation of MK gene expression, we recently found that the MK gene is a target of WT1, a Wilms' tumour-suppressor gene product, and its expression is down-regulated by WT1 (Adachi et al, 1996). This may be relevant to the frequent overexpression of MK in Wilms' tumours (Tsutsui et al, 1993). As the overexpression of MK is observed not only in

*© Cancer Research Campaign 1999*

Wilms' tumours but also in many other human carcinomas, it is likely that other oncogene or suppressor gene products may modulate MK expression.

### ACKNOWLEDGEMENTS

This work was supported by grants from the Ministry of Education, Science and Culture of Japan, the Kudo Foundation, and the Mochida Memorial Foundation for Medical and Pharmaceutical Research. We also thank Nogaki Hospital (Nagoya, Japan) for the gift of the adenoma specimens, and Kyoko Mizutani for her help with the immunostaining.

### REFERENCES

Aaronson SA (1991) Growth factors and cancer. *Science* **245**: 1146–1153

Adachi Y, Matsubara S, Pedraza C, Ozawa M, Tsutsui J, Takamatsu H, Noguchi H, Akiyama T and Muramatsu T (1996) Midkine as a novel target for the Wilms' tumor suppressor gene (WT1). *Oncogene* **13**: 2197–2203

Aridome K, Tsutsui J, Takao S, Kadomatsu K, Ozaw M, Aikou T and Muramatsu T (1995) Increased midkine gene expression in human gastrointestinal cancers. *Jpn J Cancer Res* **86**: 655–661

Aster VA and Coller FA (1954) The prognostic significance of direct extension of the colon and rectum. *Ann Surg* **139**: 846–852

Choudhuri R, Zhang HT, Dnnini S, Ziche M and Bicknell R (1997) An angiogenic role for the neurokines midkine and pleiotrophin in tumorigenesis. *Cancer Res* **57**: 1814–1819

Cross M and Dexter TM (1991) Growth factors in development, transformation, and tumorigenesis. *Cell* **64**: 271–280

Daughaday W and Deuel TF (1991) Tumour secretion of growth factors. *Endocrinol Metab Clin N Am* **20**: 539–563

Dawson T and Wynford-Thomas D (1995) Does autocrine growth factor secretion form part of a mechanism which paradoxically protects against tumour development? *Br J Cancer* **71**: 1136–1141

Garver RI Jr, Chan CS and Milner PG (1993) Reciprocal expression of pleiotrophin and midkine in normal versus malignant lung tissues. *Am J Respir Cell Mol Biol* **9**: 463–466

Garver RI Jr, Radford DM, Donis-Keller H, Wick MR and Milner PG (1994) Midkine and pleiotrophin expression in normal and malignant breast tissue. *Cancer* **74**: 1584–1590

Hughes CJ, Reed JA, Cabal R, Huvos AG, Albino AP and Schantz SP (1994) Increased expression of basic growth factor in squamous carcinogenesis of the head and neck is less prevalent following smoking cessation. *Am J Surg* **168**: 381–385

Kadomatsu K, Anzano MA, Slayter MV, Winokur TS, Smith JM and Sporn MB (1993) Expression of sulfated glycoprotein 2 is associated with carcinogenesis induced by N-nitroso-N-methylurea in rat prostate and seminal vesicle. *Cancer Res* **53**: 1480–1483

Kadomatsu K, Hagihara M, Akhter S, Fan QW, Muramatsu H and Muramatsu T (1997) Midkine induces the transformation of NIH3T3 cells. *Br J Cancer* **75**: 354–359

Kadomatsu K, Huang RP, Suganama T, Murata F and Muramatsu T (1990) A retinoic acid responsive gene MK found in the teratocarcinoma system is expressed in a spatially and temporally controlled manner during mouse embryogenesis. *J Cell Biol* **110**: 607–616

Kadomatsu K, Tomomura M and Muramatsu T (1988) cDNA cloning and sequencing of a new gene intensely expressed in early differentiation stages of embryonal carcinoma cells and in mid-gestation period of mouse embryogenesis. *Biochem Biophys Res Commun* **151**: 1312–1318

Laemmli UK (1970) Cleavage of structural proteins during assembly of the head of bacteriophage T4. *Nature* **227**: 680–685

Michikawa M, Kikuchi S, Muramatsu H, Muramatsu T and Kim SU (1993) Retinoic acid responsive gene product, Midkine, has neurotrophic functions for mouse spinal cord and dorsal root ganglion neurons in culture. *J Neurosci Res* **35**: 530–539

Muramatsu T (1993) Midkine, the product of a retinoic acid responsive gene, and pleiotrophin constitute a new protein family regulating growth and differentiation. *Int J Dev Biol* **37**: 183–188

Muramatsu T (1994) The midkine family of growth/differentiation factors. *Dev Growth Differ* **36**: 1–8

Muramatsu H and Muramatsu T (1991) Purification of recombinant midkine and examination of its biological activities: functional comparison of new heparin binding factors. *Biochem Biophys Res Commun* **177**: 652–658

Muramatsu H, Shirahama H, Yonezawa S, Maruta H and Muramatsu T (1993) Midkine, a retinoic acid-inducible growth/differentiation factor: immunochemical evidence for the function and distribution. *Dev Biol* **159**: 392–402

Nakagawara A, Milbrandt J, Muramatsu T, Deuel TF, Shao H, Cnaan A and Brodeur GM (1995) Differential expression of pleiotrophin and midkine in advanced neuroblastomas. *Cancer Res* **55**: 1792–1797

Nakajima-Iijima S, Hamada H, Reddy P and Kakunaga T (1993) Molecular structure of the human cytoplasmic beta-acint gene; interspecies homology of sequences in the introns. *Proc Natl Acad Sci USA* **82**: 6133–6137

Nurcombe V, Fraser N, Herlaar E and Heath J K (1992) MK: a pluripotential embryonic stem-cell-derived neuroregulatory factor. *Development* **116**, 1175–1183

O'Brien T, Cranston D, Fuggle S, Bicknell R and Harris AL (1996) The angiogenic factor midkine is expressed in bladder cancer, and overexpression correlates with a poor outcome in patients with invasive cancers. *Cancer Res* **56**: 2515–2518

Pitot HC, Dragan YP, Teeguarden J, Hsia S and Campbell H (1996) Quantitation of multistage carcinogenesis in rat liver. *Toxicol Pathol* **24**: 119–128

Sandgren EP, Luetteke NC, Qiu TH, Palmiter RD, Brinster RL and Lee DC (1993) Transforming growth factor α dramatically enhances oncogene-induced carcinogenesis in transgenic mouse pancreas and liver. *Mol Cell Biol* **13**: 320–330

Sporn MB and Todaro GJ (1980) Autocrine secretion and malignant transformation of cells. *N Engl J Med* **303**: 878–880

Tamano S, Merlino GT and Ward JM (1994) Rapid development of hepatic tumors in transforming growth factor α transgenic mice associated with increased cell proliferation in precancerous hepatocellular lesions initiated by N-nitrosodiethylamine and promoted by phenobarbital. *Carcinogenesis* **15**: 1791–1798

Tomomura M, Kadomatsu K, Matsubara S and Muramatsu T (1990*a*) A retinoic acid-responsive gene, MK, found in the teratocarcinoma system. *J Biol Chem* **265**: 10765–10770

Tomomura M, Kadomatsu K, Nakamoto M, Muramatsu H, Kondoh H, Imagawa K and Muramatsu T (1990*b*) A retinoic acid responsive gene, MK, produces a secreted protein with heparin binding activity. *Biochem Biophys Res Commun* **171**: 603–609

Tsutsui J, Kadomatsu K, Matsubara S, Nakagawara A, Hamanoue M, Takao S, Shimazu H, Ohi Y and Muramatsu T (1993) A new family of heparin-binding growth/differentiation factors: increased midkine expression in Wilms' tumor and other human carcinomas. *Cancer Res* **53**: 1281–1285

Unoki K, Ohba N, Arimura H, Muramatsu H and Muramatsu T (1994) Rescue of photoreceptors from the damaging effects of constant light by Midkine, a retinoic acid-responsive gene product. *Invest Ophthalmol Vis Sci* **35**: 907–915

Yang D, Alt E and Rogler CE (1993) Coordinate expression of N-myc 2 and insulin-like growth factor II in precancerous altered hepatic foci in woodchuck hepatitis virus carriers. *Cancer Res* **53**: 2020–2027

 © Cancer Research Campaign 1999
